# Effect of Co-Administration of Rivaroxaban and Clopidogrel on Bleeding Time, Pharmacodynamics and Pharmacokinetics: A Phase I Study

**DOI:** 10.3390/ph5030279

**Published:** 2012-02-24

**Authors:** Dagmar Kubitza, Michael Becka, Wolfgang Mück, Stephan Schwers

**Affiliations:** Clinical Pharmacology, Bayer Pharma AG, D-42096 Wuppertal, Germany

**Keywords:** bleeding time, clopidogrel, pharmacodynamics, pharmacokinetics, rivaroxaban

## Abstract

Dual antiplatelet therapy with acetylsalicylic acid and a thienopyridine, such as clopidogrel, is effective for the secondary prevention of cardiovascular events in patients with acute coronary syndrome, but there is still a substantial residual risk of recurrence. Although anticoagulant therapy with a vitamin K antagonist (e.g. warfarin) in conjunction with antiplatelet therapy has been shown to reduce the risk of cardiovascular events, the rates of bleeding were increased with these combination therapies; hence, triple therapy with warfarin is currently only recommended in patients at low risk of bleeding. In addition, there are other limitations associated with vitamin K antagonist therapy, including the need for routine coagulation monitoring and dose adjustment to maintain the treatment within the therapeutic range. Rivaroxaban is an oral, direct Factor Xa inhibitor; in clinical practice, it is likely that rivaroxaban will be given to patients who also receive antiplatelet therapy, such as clopidogrel. This randomized, non-blinded, three-way crossover study investigated the effect of rivaroxaban on bleeding time when co­administered with clopidogrel. In addition, the influence of clopidogrel on the safety, tolerability, pharmacodynamics and pharmacokinetics of rivaroxaban was investigated. Of 27 healthy male subjects who received a single 300 mg dose of clopidogrel, 14 were identified as clopidogrel responders and were then randomized to the following three treatments: (A) two doses of clopidogrel on two consecutive days (300 mg on day 1; 75 mg on day 2); (B) one dose of rivaroxaban (15 mg); or (C) a combination of treatments A and B (rivaroxaban given on day 2). All treatments were well tolerated. Bleeding time with co­administration of rivaroxaban and clopidogrel was significantly prolonged in four subjects, compared with either drug alone: combination treatment increased the overall least squares-means to 3.77 times baseline (90% confidence interval [CI] 2.82–4.73), compared with 1.13 times baseline (90% CI 0.17–2.09) with rivaroxaban and 1.96 times baseline (90% CI 0.10–2.91) with clopidogrel. Co-administration of clopidogrel had no significant effect on the pharmacokinetics of rivaroxaban and, when compared with rivaroxaban alone, had no further effects on Factor Xa activity or prothrombin time. Inhibition of ADP-stimulated platelet aggregation by clopidogrel was not affected by rivaroxaban. As expected, owing to the mode of action of each study drug, the results of this study demonstrated that co­administration of the Factor Xa inhibitor rivaroxaban and the antiplatelet clopidogrel increased the bleeding time in healthy subjects without affecting other pharmacokinetic or pharmacodynamic parameters of each drug.

## 1. Introduction

Arterial and venous thromboembolic disorders are associated with substantial morbidity and mortality. Acute coronary syndrome (ACS) is caused by thrombosis in the coronary arteries. Rupture of an atherosclerotic plaque triggers thrombogenesis by platelet activation and aggregation and activation of the coagulation cascade, leading to complete or partial vessel occlusion [1]. The current gold standard of care for short-term and long-term secondary prevention of cardiovascular events in patients with ACS is dual antiplatelet therapy with acetylsalicylic acid (ASA) and a thienopyridine such as clopidogrel [2,3]. Both ASA, an irreversible inhibitor of thromboxane A2 synthesis, and clopidogrel, an inhibitor of P2Y_12_ ADP platelet receptors, inhibit molecular pathways that mediate platelet activation and, therefore, prevent such adverse events [3]. However, despite the proven clinical benefit of these agents, patients remain at a substantial residual risk of recurrent cardiovascular events [4,5]. 

Arterial thrombosis involves both platelet aggregation and the activation of the coagulation cascade, providing the rationale for anticoagulant therapy in addition to antiplatelet therapy for secondary prevention of cardiovascular events in patients with ACS [1]. A number of studies have assessed the risks and benefits of warfarin therapy in addition to ASA [6,7] or dual antiplatelet therapy (ASA and clopidogrel) [8] for the prevention of cardiovascular events in patients with ACS. These studies showed an improvement in cardiovascular outcomes compared with antiplatelet therapy alone, but this improvement was accompanied by an increase in major bleeding. Currently, triple antithrombotic therapy with warfarin, ASA and clopidogrel is only recommended in patients at low risk of bleeding [8]. Warfarin is also associated with other limitations that often result in patients receiving inadequate prophylaxis or sub-optimal patient adherence. These limitations include multiple drug-drug and food-drug interactions and unpredictable responses that necessitate routine coagulation monitoring and dose adjustments to ensure that patients maintain an appropriate anticoagulation intensity [9]. 

Novel oral anticoagulants have been developed in recent years in an attempt to overcome some of the limitations associated with traditional agents (such as unfractionated heparin, low molecular weight heparins, fondaparinux and the vitamin K antagonists). These new agents, such as the direct Factor Xa inhibitors rivaroxaban and apixaban and the direct thrombin inhibitor dabigatran etexilate, have been investigated extensively in large-scale clinical trials across several indications, including ACS. Rivaroxaban has successfully completed a phase III clinical trial in patients with ACS [10]. The phase III trial of apixaban was terminated prematurely because of safety reasons [11] and a phase III trial for dabigatran etexilate in ACS has not been performed.

Rivaroxaban has shown a predictable pharmacokinetic/pharmacodynamic (PK/PD) profile, has a rapid onset of action, high oral bioavailability, few drug-drug interactions and does not require routine coagulation monitoring or dose adjustments for age, gender or body weight [12,13]. Rivaroxaban has been shown to be effective in animal models of arterial and venous thrombosis [13,14] and has demonstrated consistent efficacy and reassuring safety in large-scale clinical trials [10,15,16,17,18,19,20,21]. Rivaroxaban has gained approval for the prevention of venous thromboembolism after elective hip or knee replacement surgery in many countries worldwide. Rivaroxaban has also gained European and US approval for the prevention of stroke and systemic embolism in adult patients with non-valvular atrial fibrillation with one or more risk factors and European approval for the treatment of deep vein thrombosis (DVT) and prevention of recurrent DVT and pulmonary embolism following an acute DVT in adults.

A previous study in healthy subjects showed that ASA did not alter the PK/PD profile of rivaroxaban; these data also showed that the combination of rivaroxaban and ASA had no additional effect on platelet aggregation or bleeding time compared with ASA alone [22]. Clopidogrel is currently the most commonly used antiplatelet agent in patients with ACS, in clinical practice, and it is likely that some patients who receive rivaroxaban may also be treated with antiplatelet agents, such as clopidogrel [23].

The objectives of this study were to investigate the effect of co-administration of rivaroxaban and clopidogrel on bleeding time and platelet aggregation, and the potential influence of clopidogrel on the safety, tolerability, PD and PK of a single dose of 15 mg rivaroxaban, and vice versa, in healthy male subjects. Based on the mode of action and the characteristics of both study drugs, bleeding time was the only parameter that was expected to be affected by co-administration of rivaroxaban and clopidogrel in comparison with either drug alone.

## 2. Experimental Section

### 2.1. Subjects

This phase I study enrolled 27 healthy male subjects who were between 18 and 55 years of age, had a body mass index within the range of 18–32 kg/m^2^, a heart rate of 45–90 beats per minute, systolic blood pressure of 100–145 mmHg and diastolic blood pressure below 95 mmHg, and who had no relevant pathological changes in their electrocardiogram (ECG). Subjects were excluded if they had participated in any other clinical trial in the three months leading up to the study or had given more than 100 mL of blood in the previous four weeks or more than 500 mL of blood in the precedingthree months. Subjects were also excluded if they had any clinically relevant condition or medical history that may affect study results or if they had any medical condition that may affect their ability to participate or complete the study.

### 2.2. Study Design and Treatments

This randomized, non-blinded, single-centre, three-way crossover study was approved by the Ethics Committee of the North-Rhine Medical Council, Düsseldorf, Germany, and was conducted in accordance with the Declaration of Helsinki, the International Conference on Harmonisation Good Clinical Practice guidelines, and German drug law. The study (study number 011864) was conducted at the Pharma Center of the Institute of Clinical Pharmacology, Bayer HealthCare AG, Wuppertal, Germany.

**Figure 1:**
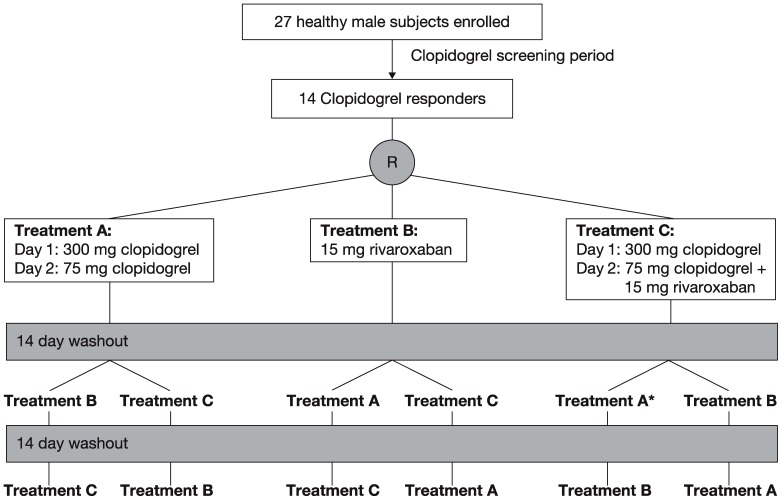
Study design of the three-way crossover study. * Treatment period during which one subject withdrew from the study owing to an adverse event.

A clopidogrel response screening period preceded the study (Figure 1). During the screening period, subjects received a single dose of 300 mg clopidogrel and platelet aggregation was measured 24 h after administration. Fourteen of the 27 subjects showed more than 40% inhibition of platelet aggregation compared with baseline and were, therefore, considered to be clopidogrel responders. The initial dose of 300 mg clopidogrel was chosen for this study because this loading dose is generally used in patients with ACS [24]. A second dose of 75 mg clopidogrel was selected because this is the standard daily dose recommended by guidelines for use in patients with a variety of relevant conditions, including atrial fibrillation, stroke and ACS [24].

The 14 clopidogrel responders were randomly assigned to one of the following three treatments with a washout phase of about 14 days between treatments (Figure 1). Treatment A consisted of 300 mg clopidogrel on day 1 and 75 mg clopidogrel on day 2. Treatment B consisted of a single dose of 15 mg rivaroxaban. Treatment C combined treatments A and B, with rivaroxaban given on day 2. Subjects were hospitalized on the evening before treatment was started in the morning and they stayed on the study ward for 3 days (treatment A), 2 days (treatment B) and 5 days (treatment C) after the first drug dose, and were discharged thereafter if there were no medical objections. The study ended with the final assessments approximately 1 week after the last treatment. All treatments were administered after 10 h of fasting at 08:00. Lunch was scheduled 4 h after tablet intake.

Rivaroxaban, at doses between 5 mg and 80 mg, has been shown to have relevant PD effects [25,26,27]. Based on these results, a dose of 15 mg rivaroxaban was chosen as a suitable dose for this study. More recently, daily doses of 5–20 mg have been shown to be clinically efficacious in phase II and phase III trials [15,16,17,18,19,20,21,27,28].

### 2.3. Safety and Tolerability

Safety and tolerability were assessed subjectively and objectively. Subjective assessment was obtained by asking the subjects non-leading questions about the occurrence of any adverse events or by spontaneous reporting of adverse events. Adverse events were classified according to their degree of severity. Objective tolerability was evaluated by monitoring cardiovascular parameters including heart rate, blood pressure and ECG parameters. In addition, blood tests, clinical chemistry, urine test and drug screening were part of the objective assessment.

### 2.4. Bleeding Time

Bleeding time tests were performed in accordance with the protocol reported by the International Committee on Standardization of the Bleeding Time [29]. Bleeding time measurements depend on the method and on who carries out the procedure; therefore, results vary greatly between studies. To reduce variability, one study nurse was specifically trained in the procedure of the test before this study. The same study nurse carried out all measurements at all time points for each subject. Briefly, the bleeding time test was performed on the lateral aspect of the volar surface of the forearm 3–5 cm distal to the elbow crease in an area devoid of hair, scars, bruises or surface veins. A sphygmomanometer on the upper arm was inflated to 40 mmHg for 30 seconds before the incision was made and maintained at 40 mmHg until the end of the procedure. The bleeding time incision was made by placing a Surgicutt Adult^®^ (ITC, Edison, NJ, USA) device (used in accordance with the recommendations of the manufacturer, as specified in the package insert) gently against the forearm, perpendicular (vertical) to the elbow crease. After the incision, the drops of blood flowing from the wound were wicked with filter paper (Rundfilter by Schleich und Schuell, diameter 70 mm) every 30 seconds until bleeding ceased, with care taken not to touch the incision or dislodge the developing platelet plug. The bleeding time was measured in seconds as the total duration of blood flow from the wound. A closure was placed across the incision, and the subjects were asked not to remove it for 24 h.

### 2.5. Pharmacodynamic Parameters

The effects of rivaroxaban and clopidogrel on Factor Xa activity, prothrombin time (PT), activated partial thromboplastin time (aPTT) and HepTest were assessed, as described previously [26]. Factor Xa activities above 0.1 IU/mL (the lower limit of quantification) were determined with a precision of 3.8–6.6% and an accuracy of 96–114%. PT (assessed using freeze-dried thromboplastin from rabbit brain (Neoplastine Plus; Roche Diagnostics, Mannheim, Germany), aPTT [assessed using a kaolin-activated test (Roche Diagnostics)], and HepTest^®^ (Haemachem, St. Louis, MO, USA) were measured with a ball coagulometer (KC 10, Amelung, Germany) in accordance with the manufacturer’s instructions. Blood samples were taken at the time of administration of rivaroxaban and again after 0.5, 1, 2, 3, 4, 6, 8, 12, 15, 24 and 48 h (treatment B). When clopidogrel and rivaroxaban were co-administered on day 2 of treatment C, blood samples were collected at the same time points and after 96 h. When only clopidogrel was administered (treatment A), blood samples were taken after 4, 8, 24 and 48 h. Blood samples were centrifuged to obtain plasma samples, which were then frozen and stored below −15 °C until analysis.

#### 2.5.1. Platelet Aggregation

Platelet aggregation was determined by the Born method just before and 4 h after study drug administration [30]. This method is based on the turbidimetric determination of cell suspensions. Because platelet-enriched plasma possesses a minor permeability for long-wave light (whereas plasma poor in platelets lets light pass through without obstruction), the percentage of light permeability of plasma can be used as a measure for platelet aggregation. Measurement was performed at 650 nm. The individual response of changes of platelet aggregation to baseline was determined using 29 µM ADP in addition to an optimized individual activating regimen using ADP. The light transmission of each aggregated sample is reported in relation to the light transmission of the individual platelet-enriched plasma sample (0% light transmission) and the corresponding individual platelet-poor plasma sample (prepared by centrifugation; 100% light transmission). Within 5 minutes, the maximum aggregation and the maximum gradient were evaluated against the different ADP concentrations that were used for stimulation.

#### 2.5.2. Platelet Activation Markers

The relative changes to baseline at 4 h after drug administration of the platelet activation markers membrane glycoprotein receptor GPIIb/IIIa, P-selectin and annexin V were determined by flow cytometry [31]. To assure uniformity of assays and to minimize sample manipulation, blood samples were mixed gently and processed without delay. Platelet-rich plasma was prepared by slow centrifugation, and platelets were labelled with monoclonal antibodies against GPIIb/IIIa (CD41a), P­selectin (CD62p) and annexin V protein. Samples were analysed using a COULTER^®^ EPICS^®^ XL™ flow cytometer (Beckman Coulter, Inc.; Brea, CA, USA). The average immunofluorescence of the total population or the fraction of activated events was determined and compared with individual control samples before treatment.

### 2.6. Pharmacokinetic Parameters

Blood samples for analyses of the PK parameters of rivaroxaban (area under the concentration-time curve [AUC], maximum plasma concentration [C_max_], terminal half-life [t_½_] were taken at the time of administration of rivaroxaban and again after 0.5, 1, 2, 3, 4, 6, 8, 12, 15, 24 and 48 h for treatment B and treatment C). PK parameters were determined by non-compartmental analysis using KINCALC^®^ (Bayer HealthCare AG, Wuppertal, Germany). Quantitative analysis of rivaroxaban in plasma was performed using a fully validated assay. Briefly, concentrations of rivaroxaban were determined after solid/liquid extraction by high-performance liquid chromatography coupled with a tandem mass spectrometer. A close chemical analogue of rivaroxaban, BAY 60-4758 (5-chloro-N-({3­[3,5-dimethyl-4-(3-oxomorpholin-4-yl)phenyl]-2-oxo-1,3-oxazolidin-5-yl}methyl)thiophene-2-carboxamide), was used as the internal standard. Prior to the high-performance liquid chromatography analysis, rivaroxaban and the internal standard were extracted from the matrix by solid phase extraction using C18 cartridges. The calibration range of the procedure was from 0.5 µg/L (lower limit of quantification) to 500 µg/L. Quality control samples in the concentration range from 1.35 µg/L to 266 µg/L were determined with an accuracy of 96.3–98.2% and a precision of 3.45–7.91% (n = 15 each).

### 2.7. Statistical Analysis

Statistical evaluations were performed with the use of the SAS^®^ software package version 8.2 at the Department of Global Pharmacometrics (Bayer HealthCare AG, Wuppertal, Germany). Bleeding time results and platelet aggregation results were analysed for each subject 4 h after drug administration using descriptive statistical methods. Student’s paired *t* tests were used to compare treatments for these parameters, and 90% confidence intervals (CIs) for the difference were calculated.

The primary PK parameters AUC and C_max_ of rivaroxaban were analysed assuming log-normally distributed data. The logarithms of AUC and C_max_ were analysed by analysis of variance (ANOVA) including sequence, subject (sequence), period and treatment effects. Based on these analyses, a point estimator (least squares-means) and 90% CI for the ratio (rivaroxaban + clopidogrel)/(rivaroxaban alone) was calculated by re-transformation of the logarithmic data using the intraindividual standard deviation of the ANOVA.

## 3. Results

This study was conducted between 2 September 2005 (first screening) and 5 December 2005 (last assessment). Of the 27 healthy male subjects enrolled in this study, 14 were identified as clopidogrel responders in the screening period and were randomly assigned to one of the six possible treatment sequences. The 14 subjects had a mean age of 33.6 years (range 26–43 years), weight of 78.4 ± 10.2 kg and body mass index of 24.6 ± 2.7 kg/m^2^. One subject who had completed the combined treatment withdrew during the second treatment period (clopidogrel alone) because of febrile infection and, therefore, was not included in the PK and PD analyses.

### 3.1. Safety and Tolerability

A total of 24 treatment-emergent adverse events were reported by 11 of the 14 responders, and headache was the most common type of adverse event (7 events). The intensity of the events was always mild, and all events were resolved by the time of study completion. One adverse event, febrile infection, led to the withdrawal from the study of one subject (Figure 1), but the occurrence of this adverse event was not considered to be drug related. Therefore, the data suggest that a single dose of 15 mg rivaroxaban is safe and well tolerated alone and in combination with clopidogrel.

### 3.2. Bleeding Time

**Figure 2:**
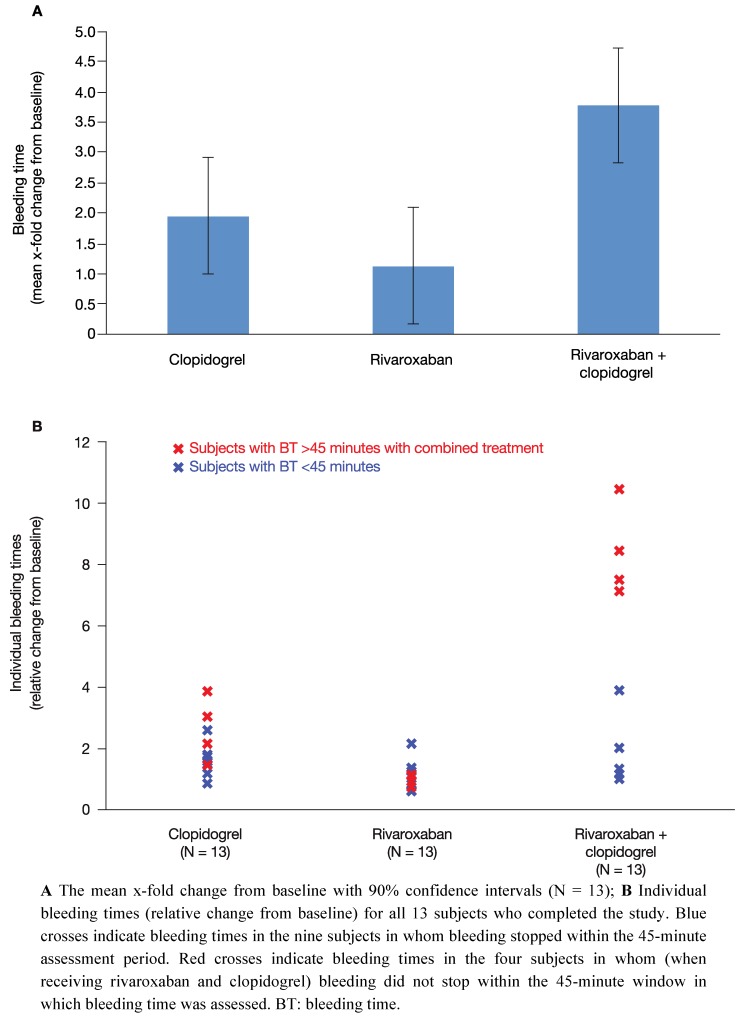
Bleeding time. The effect of clopidogrel and rivaroxaban alone and in combination on bleeding time.

All 14 subjects showed normal levels of standard haematological parameters throughout the study period, including haematocrit, haemoglobin and cell counts of different types of blood cells. At baseline, mean bleeding time was 6.99 ± 2.20 minutes (range 4.30–10.3 minutes) and was within the normal range (2–8 minutes). Bleeding time was measured 4 h after the last drug administration for each treatment. Compared with baseline, a single dose of 15 mg rivaroxaban (treatment B) resulted in a 1.1-fold least squares-mean relative change in bleeding time (90% CI 0.17–2.09) (Figure 2A). Administration of clopidogrel alone on two consecutive days (treatment A: 300 mg on day 1; 75 mg on day 2) increased the bleeding time by 2.0-fold (90% CI 1.00–2.91) (Figure 2A). These data indicate that monotherapy with rivaroxaban had no relevant effect on bleeding time and monotherapy with clopidogrel had only a borderline significant effect (two-fold increase in bleeding time). 

After co-administration of rivaroxaban and clopidogrel (treatment C) bleeding time was assessed 4 h after drug administration on day 2. There was a significant 3.8-fold increase in bleeding time relative to baseline (90% CI 2.82–4.73) (Figure 2A). The difference in least squares-mean bleeding time between clopidogrel only and the combination treatment with clopidogrel and rivaroxaban (relative changes to baseline) was 1.8-fold (90% CI 0.47–3.17). With regards to prolonged bleeding time after co-administration, two subgroups of subjects could be distinguished. One subgroup (9 of 13 clopidogrel responders; blue crosses, Figure 2B) showed a mean 2 to 3-fold prolongation of bleeding time relative to baseline (Figure 2B). This is only a slight increase in bleeding time compared with the 2-fold increase in bleeding time with clopidogrel alone. The other subgroup was composed of four subjects who did not stop bleeding within the 45-minute time window in which bleeding time was assessed (red crosses, Figure 2B). It is noteworthy that bleeding time in three of these four subjects had also exceeded 45 minutes when bleeding time was assessed during the screening period.

In summary, mean bleeding time after combined treatment was significantly prolonged compared with each of the single treatments. Bleeding time doubled when rivaroxaban was administered together with clopidogrel compared with clopidogrel alone, indicating an additive effect. However, there was considerable variation between subjects; a significant prolongation of bleeding time beyond that of clopidogrel alone was only observed in approximately one-third of subjects. Furthermore, the substantially increased bleeding time in these four subjects did not correlate with any of the platelet aggregation markers assessed in this study.

### 3.3. Pharmacodynamics

**Figure 3:**
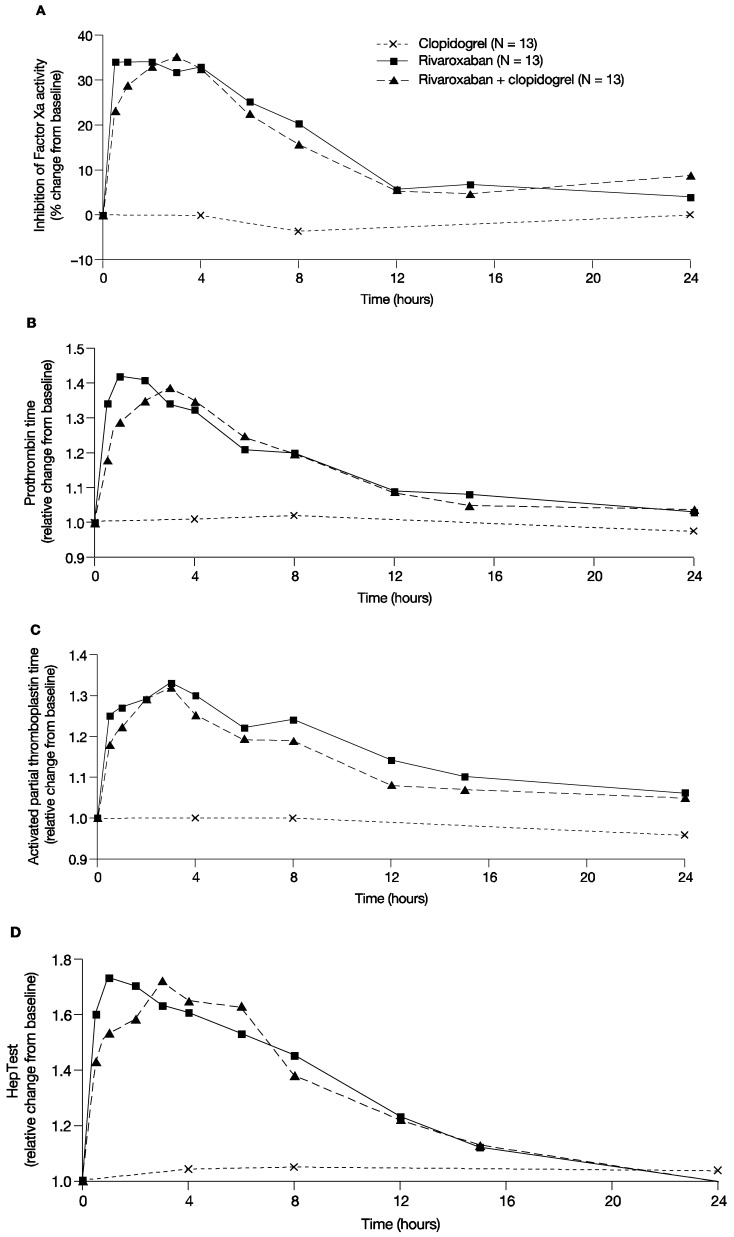
Pharmacodynamic parameters. The effect of rivaroxaban and clopidogrel alone and in combination on (**A**) the median percentage change from baseline of the inhibition of Factor Xa activity over time; (**B**) Median relative change from baseline of prothrombin time over time; (**C**) Median relative change from baseline of the activated partial thromboplastin time over time; (**D**) Median relative change from baseline of the HepTest over time.

In line with previous studies, rivaroxaban showed a rapid inhibition of Factor Xa activity; the median value of the Factor Xa activity at 4 h after administration of 15 mg rivaroxaban was 0.57 U/mL. After co-administration of clopidogrel and rivaroxaban, the median value of Factor Xa activity was 0.61 U/mL and, therefore, similar to rivaroxaban alone. Clopidogrel only had no effect on Factor Xa activity; 4 h after clopidogrel administration the median value of Factor Xa activity was 0.9 U/mL. The median percentage change from baseline for Factor Xa inhibition with a single dose of 15 mg rivaroxaban was between 31.8% and 34.1% for the first 4 h post administration and returned to values within 10% of baseline at 12 h after administration. Clopidogrel alone did not affect Factor Xa activity, and co-administration of clopidogrel with rivaroxaban had no additional effect on Factor Xa activity compared with rivaroxaban alone (Figure 3A). Similarly, monotherapy with rivaroxaban prolonged PT for the first 4 h after administration with a median relative change from baseline between 1.31 and 1.42, and values returned to values within 10% of baseline at 12 h after administration (Figure 3B). Co-administration of clopidogrel and rivaroxaban showed similar results, and clopidogrel alone did not change PT compared with baseline (Figure 3B). Similar results were also observed for aPTT and HepTest (Figure 3C,D).

In summary, administration of rivaroxaban alone had the expected effect on Factor Xa, PT, aPTT and HepTest. Combined treatment had no additional effect on these parameters and clopidogrel alone did not affect Factor Xa activity, PT, aPTT and HepTest, versus treatment with rivaroxaban alone.

### 3.4. Platelet Aggregation

**Figure 4:**
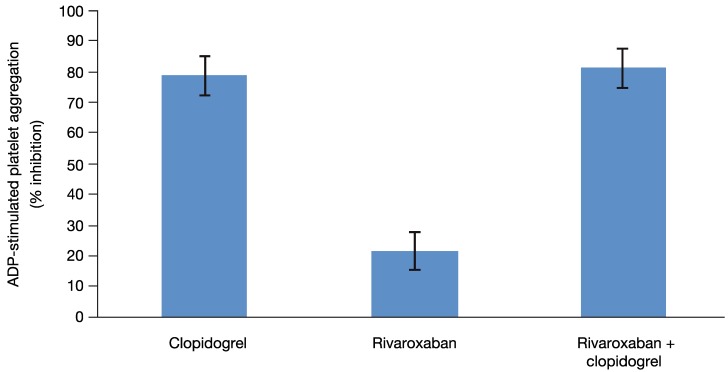
Platelet aggregation. The effect of clopidogrel and rivaroxaban alone and in combination on 29 µM ADP-stimulated platelet aggregation. Least squares-mean values of the percent inhibition and 2-sided 90% confidence intervals are shown, as assessed by the ANOVA method.

Because all 13 subjects included in this analysis had been identified as clopidogrel responders on the basis of the platelet aggregation test, clopidogrel alone had the expected effect on platelet aggregation. In addition, as expected, platelet aggregation was not affected by rivaroxaban alone when measured with individually optimized concentration of ADP or with a fixed concentration (29 µM) of ADP (Figure 4). The median relative change from baseline for platelet aggregation was 0.9 with optimized ADP and 1.1 with 29 µM ADP. Both methods showed that platelet aggregation was inhibited to a similar extent by either clopidogrel alone (median relative change from baseline 0.249 with optimized ADP and 0.340 with 29 µM ADP) or by the combined treatment (median relative change from baseline 0.282 with optimized ADP and 0.325 with 29 µM ADP). Therefore, rivaroxaban alone or in combination with clopidogrel had no effect on platelet aggregation.

Similarly, administration of rivaroxaban alone had no relevant effect on the platelet activation markers. After administration of clopidogrel either alone or in combination with rivaroxaban, a pronounced decrease of the platelet activation markers was observed, but rivaroxaban had no additional effect.

### 3.5. Pharmacokinetic Parameters

After administration of rivaroxaban in combination with clopidogrel, geometric means for AUC and C_max_ were similar to the geometric means after administration of rivaroxaban alone. The 90% CIs for the ratios of AUC and C_max_ (0.85–1.12 for AUC and 0.81–1.04 for C_max_) were fully included in the generally accepted range for the assumption of equal bioavailability (0.80–1.25) (Table 1). In addition, co-administration of rivaroxaban and clopidogrel did not change other PK parameters of rivaroxaban compared with the administration of rivaroxaban alone.

**Table 1:** Pharmacokinetic parameters in plasma following a single oral dose of 15 mg rivaroxaban alone or in combination with 75 mg clopidogrel [geometric mean values/%CV (range), N = 13]. AUC: area under the concentration-time curve; C_max_: maximum plasma concentration; t_½_: terminal half-life.

Parameter	Unit	N	Rivaroxaban	N	Rivaroxaban + clopidogrel
AUC	μg•h/L	13	1,477/29.0 (934–2,579)	13	1,432/37.8 (684–2,797)
C_max_	μg/L	13	168/33.4 (106–274)	13	153/28.0 (96.0–213)
t_½_	h	13	9.00/27.4 (5.99–14.6)	13	8.81/24.0 (5.45–13.2)

## 4. Discussion

This study showed that co-administration of rivaroxaban and clopidogrel resulted in a 3.77-fold increase in bleeding time when compared with baseline and a doubling of bleeding time when compared with clopidogrel alone. These results were mainly owing to a significantly increased bleeding time in one-third of subjects (Figure 2B). An increase in bleeding time is a well-known phenomenon with combined antithrombotic therapy. Previous studies have shown increases in bleeding time after co­administration of ASA and clopidogrel. A phase I study in healthy subjects reported a prolongation from 7.6 ± 3.4 minutes to 17.5 ± 8.6 minutes (*p* < 0.05) when 75 mg clopidogrel was administered in addition to 150 mg ASA on two consecutive days [32]. The effect on bleeding time was even more pronounced with 300 mg clopidogrel in addition to ASA (24.9 ± 8.5 minutes; *p* < 0.05) [32]. Of note, in the study by Payne *et al*., three of the seven subjects showed a significant prolongation of bleeding time that exceeded the 30-minute assessment window after receiving 150 mg ASA and 300 mg clopidogrel [32]. Another phase I study by Wåhlander *et al.* showed that bleeding time was prolonged 6.4-fold when clopidogrel was co-administered with ASA in healthy male subjects [33]. Despite the fact that results from clinical trials also reported a significant increase in risk of bleeding with ASA and clopidogrel, efficacy for the prevention of adverse cardiovascular events was enhanced in patients after ACS, which led to an overall positive benefit-risk assessment and the acceptance of the combination of ASA and clopidogrel as the current standard of care for the prevention of atherothrombotic events in patients after ACS [2,4,5]. It is important to note that bleeding time is not predictive of bleeding risk in individual patients receiving antithrombotic agents [34,35]; however, increased bleeding time observed in a clinical study setting may indicate a potential for a higher risk of bleeding in the overall population. Because the results of this phase I study with rivaroxaban were similar to two published studies that combined ASA and clopidogrel [32,33] – a treatment regimen with an accepted, positive benefit-risk profile – it was deemed to be justified to progress the combination of rivaroxaban and clopidogrel into phase II and later phase III under careful assessment of both safety and efficacy.

Co-administration of rivaroxaban and antiplatelet agents has been investigated in a phase II (ATLAS ACS TIMI 46) and a phase III study (ATLAS ACS 2 TIMI 51) in patients with ACS [10,21], ATLAS ACS TIMI 46 showed a dose-dependent increase in bleeding in the rivaroxaban groups (5–20 mg), but major ischaemic outcomes were reduced in the rivaroxaban group. Low doses of rivaroxaban were selected for the ATLAS ACS 2 TIMI 51 study, which demonstrated a significantly reduced risk of death from cardiovascular causes, myocardial infarction or stroke with rivaroxaban compared with placebo [10,36]. Compared with placebo, there was no significant increase in fatal bleeding in patients receiving rivaroxaban, but rates of major bleeding and intracranial haemorrhage were increased in the rivaroxaban groups [10]. Studies investigating apixaban (phase III, APPRAISE-2) and dabigatran (phase II, RE-DEEM) in addition to antiplatelet therapy in patients with ACS have also shown increases in major bleeding events without reducing the rates of recurrent ischaemic events [11,37]. These results clearly show that there is an increase in the risk of bleeding when combining anticoagulants (such as Factor Xa inhibitors or direct thrombin inhibitors) with antiplatelet therapy, which is to be expected given the mode of action of these drugs.

The doubling of bleeding time seen with co-administration of rivaroxaban and clopidogrel compared with clopidogrel only in this study seems to be generally in line with the findings of the ATLAS ACS 2 TIMI 51 study, which reported an increased risk of major bleeding in patients who received rivaroxaban in addition to antiplatelet therapy. However, it is important to carefully assess both safety and efficacy to determine the usefulness of a treatment regimen. 

## 5. Conclusions

This phase I mechanistic study in healthy volunteers was designed to assess the potential PK and PD interactions between rivaroxaban and clopidogrel. Based on the mechanism of action of both drugs, it was expected that the co-administration of rivaroxaban (15 mg) and clopidogrel would not show any effect on the PK or PD parameters. In contrast, bleeding time could potentially be affected by both drugs. The study showed that bleeding time was significantly prolonged by the combination of rivaroxaban and clopidogrel in approximately one-third of subjects. Although bleeding time does not correlate with the risk of bleeding in an individual patient, it may indicate an overall increased risk of bleeding for a population. It is noteworthy that phase I studies that investigated the effect of the combined administration of ASA and clopidogrel showed similar results. Although clinical trials have indicated an increased risk of bleeding for the combination of ASA and clopidogrel, the enhanced efficacy led to a wide acceptance of this treatment regimen in patients with ACS. The results of this study do not preclude co-administration of rivaroxaban and clopidogrel if the benefit-risk for each individual patient is assessed and bleeding events are monitored carefully.

## References

[1] Turpie A.G.G., Esmon C. (2011). Venous and arterial thrombosis – pathogenesis and the rationale for anticoagulation. Thromb. Haemost..

[2] Anderson J.L., Adams C.D., Antman E.M., Bridges C.R., Califf R.M., Casey D.E., Chavey W.E., Fesmire F.M., Hochman J.S., Levin T.N. (2007). ACC/AHA 2007 guidelines for the management of patients with unstable angina/non ST-elevation myocardial infarction. A Report of the American College of Cardiology/American Heart Association Task Force on Practice Guidelines (Writing Committee to Revise the 2002 Guidelines for the Management of Patients With Unstable Angina/Non ST-Elevation Myocardial Infarction). Circulation.

[3] Hamm C.W., Bassand J.P., Agewall S., Bax J., Boersma E., Bueno H., Caso P., Dudek D., Gielen S., Huber K. (2011). ESC Guidelines for the management of acute coronary syndromes in patients presenting without persistent ST-segment elevation: The Task Force for the management of acute coronary syndromes (ACS) in patients presenting without persistent ST-segment elevation of the European Society of Cardiology (ESC). Eur. Heart J..

[4] Yusuf S., Zhao F., Mehta S.R., Chrolavicius S., Tognoni G., Fox K.K. (2001). Effects of clopidogrel in addition to aspirin in patients with acute coronary syndromes without ST-segment elevation. N. Engl. J. Med..

[5] Chen Z.M., Jiang L.X., Chen Y.P., Xie J.X., Pan H.C., Peto R., Collins R., Liu L.S. (2005). Addition of clopidogrel to aspirin in 45,852 patients with acute myocardial infarction: Randomised placebo-controlled trial. Lancet.

[6] Rothberg M.B., Celestin C., Fiore L.D., Lawler E., Cook J.R. (2005). Warfarin plus aspirin after myocardial infarction or the acute coronary syndrome: Meta-analysis with estimates of risk and benefit. Ann. Intern. Med..

[7] Andreotti F., Testa L., Biondi-Zoccai G.G., Crea F. (2006). Aspirin plus warfarin compared to aspirin alone after acute coronary syndromes: An updated and comprehensive meta-analysis of 25,307 patients. Eur. Heart J..

[8] Zhao H.J., Zheng Z.T., Wang Z.H., Li S.H., Zhang Y., Zhong M., Zhang W. (2011). “Triple therapy” rather than “triple threat”: A meta-analysis of the two antithrombotic regimens after stent implantation in patients receiving long-term oral anticoagulant treatment. Chest.

[9] Ansell J., Hirsh J., Hylek E., Jacobson A., Crowther M., Palareti G. (2008). Pharmacology and management of the vitamin K antagonists: American College of Chest Physicians evidence-based clinical practice guidelines (8th Edition). Chest.

[10] Mega J.L., Braunwald E., Wiviott S.D., Bassand J.P., Bhatt D.L., Bode C., Burton P., Cohen M., Cook-Bruns N., Fox K.A. (2012). Rivaroxaban in patients with a recent acute coronary syndrome. N. Engl. J. Med..

[11] Alexander J.H., Lopes R.D., James S., Kilaru R., He Y., Mohan P., Bhatt D.L., Goodman S., Verheugt F.W., Flather M. (2011). Apixaban with antiplatelet therapy after acute coronary syndrome. N. Engl. J. Med..

[12] (2011). Bayer Pharma AG. Xarelto^®^ (rivaroxaban) Summary of Product Characteristics. http://www.xarelto.com/html/downloads/Xarelto_Summary_of_Product_Characteristics_Dec2011.pdf.

[13] Perzborn E., Roehrig S., Straub A., Kubitza D., Misselwitz F. (2011). The discovery and development of rivaroxaban, an oral, direct Factor Xa inhibitor. Nat. Rev. Drug Discov..

[14] Perzborn E., Strassburger J., Wilmen A., Pohlmann J., Roehrig S., Schlemmer K.H., Straub A. (2005). *In vitro* and *in vivo* studies of the novel antithrombotic agent BAY 59-7939 - an oral, direct Factor Xa inhibitor. J. Thromb. Haemost..

[15] Eriksson B.I., Borris L.C., Friedman R.J., Haas S., Huisman M.V., Kakkar A.K., Bandel T.J., Beckmann H., Muehlhofer E., Misselwitz F. (2008). Rivaroxaban *versus* enoxaparin for thromboprophylaxis after hip arthroplasty. N. Engl. J. Med..

[16] Kakkar A.K., Brenner B., Dahl O.E., Eriksson B.I., Mouret P., Muntz J., Soglian A.G., Pap A.F., Misselwitz F., Haas S. (2008). Extended duration rivaroxaban *versus* short-term enoxaparin for the prevention of venous thromboembolism after total hip arthroplasty: A double-blind, randomised controlled trial. Lancet.

[17] Lassen M.R., Ageno W., Borris L.C., Lieberman J.R., Rosencher N., Bandel T.J.,  Misselwitz F., Turpie A.G.G., RECORD3 Investigators (2008). Rivaroxaban *versus* enoxaparin for thromboprophylaxis after total knee arthroplasty. N. Engl. J. Med..

[18] Turpie A.G.G., Lassen M.R., Davidson B.L., Bauer K.A., Gent M., Kwong L.M., Cushner F.D., Lotke P.A., Berkowitz S.D., Bandel T.J. (2009). Rivaroxaban *versus* enoxaparin for thromboprophylaxis after total knee arthroplasty (RECORD4): A randomised trial. Lancet.

[19] The EINSTEIN Investigators (2010). Oral rivaroxaban for symptomatic venous thromboembolism. N. Engl. J. Med..

[20] Patel M.R., Mahaffey K.W., Garg J., Pan G., Singer D.E., Hacke W., Breithardt G., Halperin J.L., Hankey G.J., Piccini J.P. (2011). Rivaroxaban versus warfarin in nonvalvular atrial fibrillation. N. Engl. J. Med..

[21] Mega J.L., Braunwald E., Mohanavelu S., Burton P., Poulter R., Misselwitz F., Hricak V., Barnathan E.S., Bordes P., Witkowski A. (2009). Rivaroxaban versus placebo in patients with acute coronary syndromes (ATLAS ACS-TIMI 46): A randomised, double-blind, phase II trial. Lancet.

[22] Kubitza D., Becka M., Mueck W., Zuehlsdorf M. (2006). Safety, tolerability, pharmacodynamics, and pharmacokinetics of rivaroxaban—an oral, direct Factor Xa inhibitor—are not affected by aspirin. J. Clin. Pharmacol..

[23] Mega J.L., Simon T., Collet J.P., Anderson J.L., Antman E.M., Bliden K., Cannon C.P., Danchin N., Giusti B., Gurbel P. (2010). Reduced-function CYP2C19 genotype and risk of adverse clinical outcomes among patients treated with clopidogrel predominantly for PCI: A meta-analysis. JAMA.

[24] (2011). Sanofi Pharma Bristol-Myers Squibb SNC. Plavix (clopidogrel) Summary of Product Characteristics. http://www.ema.europa.eu/docs/en_GB/document_library/EPAR_-_Product_Information/human/000174/WC500042189.pdf.

[25] Kubitza D., Becka M., Voith B., Zuehlsdorf M., Wensing G. (2005). Safety, pharmacodynamics, and pharmacokinetics of single doses of BAY 59-7939, an oral, direct Factor Xa inhibitor. Clin. Pharmacol. Ther..

[26] Kubitza D., Becka M., Wensing G., Voith B., Zuehlsdorf M. (2005). Safety, pharmacodynamics, and pharmacokinetics of BAY 59-7939—an oral, direct Factor Xa inhibitor—after multiple dosing in healthy male subjects. Eur. J. Clin. Pharmacol..

[27] Turpie A.G.G., Fisher W.D., Bauer K.A., Kwong L.M., Irwin M.W., Kälebo P., Misselwitz F., Gent M., ODXIa-Knee Study Group (2005). BAY 59-7939: An oral, direct Factor Xa inhibitor for the prevention of venous thromboembolism in patients after total knee replacement. A phase II dose-ranging study. J. Thromb. Haemost..

[28] Eriksson B.I., Borris L., Dahl O.E., Haas S., Huisman M.V., Kakkar A.K., Misselwitz F., Kälebo P., ODIXa-HIP Study Investigators (2006). Oral, direct Factor Xa inhibition with BAY 59-7939 for the prevention of venous thromboembolism after total hip replacement. J. Thromb. Haemost..

[29] Mielke C.H. (1984). Measurement of the bleeding time. Thromb. Haemost..

[30] Born G.V., Cross M.J. (1963). The aggregation of blood platelets. J. Physiol..

[31] Tomer A. (2004). Platelet activation as a marker for *in vivo* prothrombotic activity: Detection by flow cytometry. J. Biol. Regul. Homeost. Agents.

[32] Payne D.A., Hayes P.D., Jones C.I., Belham P., Naylor A.R., Goodall A.H. (2002). Combined therapy with clopidogrel and aspirin significantly increases the bleeding time through a synergistic antiplatelet action. J. Vasc. Surg..

[33] Wåhlander K., Eriksson-Lepkowska M., Nyström P., Eriksson U.G., Sarich T.C., Badimon J.J., Kalies I., Elg M., Bylock A. (2006). Antithrombotic effects of ximelagatran plus acetylsalicylic acid (ASA) and clopidogrel plus ASA in a human *ex vivo* arterial thrombosis model. Thromb. Haemost..

[34] de Caterina R., Lanza M., Manca G., Strata G.B., Maffei S., Salvatore L. (1994). Bleeding time and bleeding: An analysis of the relationship of the bleeding time test with parameters of surgical bleeding. Blood.

[35] Barber A., Green D., Galluzzo T., Ts'ao C.H. (1985). The bleeding time as a preoperative screening test. Am. J. Med..

[36] Gibson C.M., Mega J.L., Burton P., Goto S., Verheugt F., Bode C., Plotnikov A., Sun X., Cook-Bruns N., Braunwald E. (2011). Rationale and design of the Anti-Xa Therapy to Lower cardiovascular events in Addition to standard therapy in Subjects with Acute Coronary Syndrome-Thrombolysis in Myocardial Infarction 51 (ATLAS-ACS 2 TIMI 51) trial: A randomized, double-blind, placebo-controlled study to evaluate the efficacy and safety of rivaroxaban in subjects with acute coronary syndrome. Am. Heart J..

[37] Oldgren J., Budaj A., Granger C.B., Khder Y., Roberts J., Siegbahn A., Tijssen J.G., van de Werf F., Wallentin L., RE-DEEM Investigators (2011). Dabigatran *vs.* placebo in patients with acute coronary syndromes on dual antiplatelet therapy: A randomized, double-blind, phase II trial. Eur. Heart J..

